# Culture and history of Chinese science

**DOI:** 10.1093/nsr/nwz074

**Published:** 2019-07-17

**Authors:** Mu-ming Poo

**Affiliations:** Editor-in-Chief of NSR

There has been a remarkable event in the Chinese academia in the past months that deserves attention. China Association of Science and Technology (the largest S&T organization in China comprising more than 200 national member societies) and Peking University have jointly founded an ‘Institute of Chinese Scientific Culture’. This was followed by the establishment in Peking University of a new department of ‘History of Science, Technology and Medicine’. These institutions are marked by their unique cross-disciplinary nature in research and education that encompasses all areas of sciences and humanities, and their goal of nurturing a new brand of intellectuals that China urgently needs for future development in science and society.

Exactly one century ago, on 4 May 1919, the faculty and students of Peking University sparked a new wave of modernization in China. Branded as ‘the May Fourth Movement’ [1] or ‘New Cultural Movement’, this wave was triggered by a political event—a spontaneous students’ demonstration against the government's ineffective response to the Treaty of Versailles that granted post-war transfer of the territory and concession rights in China from Germany to Japan. The undercurrent, however, was the growing demand of the Chinese intelligentsia for political and cultural modernization of China. Mr. De (‘democracy’) and Mr. Sai (‘science’) were to be invited into China, although exactly what they should look like was hotly debated. Forbearers of the May Fourth Movement were inspired by either Bolshevik revolution or parliamentary democracy of the West. None foresaw that it would eventually be a socialist democracy with stability and continuity, together with a Western-style market economy that has achieved, within a quarter of a century social transformation on a scale the world has never seen before. As President Xi Jinping recently summarized: ‘The May Fourth Movement was an anti-imperialism and anti-feudalism revolution, and a movement of enlightenment and new culture that powerfully promoted the will and confidence of Chinese people to realize national rejuvenation.’ The future of Chinese science will evolve within the context of this continuing will of the Chinese people for national rejuvenation.

One would thus wonder what could the studies of ‘Chinese Scientific Culture’ entail. Science represents human's quest for truth underlying natural phenomena, with universal values and norms that should not be biased by cultural differences. On the other hand, cultural and societal factors could influence how universal values and norms are perceived, accepted and practiced. Modern science, defined by Joseph Needham as ‘a combination of mathematized hypotheses about natural phenomena with relentless experimentation’ [2], originated from Europe, under a cultural, social and economic backdrop that was distinct from that in China. How would the Chinese cultural tradition affect the instillation of the values and norms of modern science in China? Would the current social and economic backdrop in China influence the development of Chinese science?

In the concluding volume of the monumental series of monographs on ‘Science and Civilization in China’, Joseph Needham offered his answer to the famous question of ‘Why modern science did not emerge in China’. He proposed that the ‘bureaucratic feudalism’ had prevented the emergence of modern science in China [2]. Would such an element in the cultural tradition impede scientific development in modern China? It is often said that science is distinct from technology and that ‘pure’ curiosity-driven scientific pursuit is the fountain for new technologies. However, in both China and the West, utilitarian motives had historically played a major role in the development of astronomy, mathematics, chemistry and biology. The dualistic view of science and technology inherent in the pro and con arguments for basic vs. applied research is quite alien to Chinese civilization, as amply demonstrated in Needham's volumes. The utilitarian outlook of science is thus likely to influence future scientific development in China.

Less deliberated but equally important is Needham's second question—‘Why, between the first century BC and the fifteenth century AD, was Chinese civilization much more efficient than occidental in gaining natural knowledge, and in applying it to practical human needs?’ [2] Perhaps the most interesting issue for the studies of Chinese scientific culture is whether, because of her unique history and culture, Chinese science could again make unique contribution to humanity in the future. There are a wide range of urgent global problems—from environmental pollution, global warming and energy crisis, to food distribution, and infectious and chronic diseases—that remain unsolved despite notable advances in S&T in the West. As China is the most populous country and now the second largest economy, the evolution of Chinese scientific culture is bound to be a critical factor in shaping the future of mankind.
